# Nature’s sophisticated architecture: a strategic approach for protein-polysaccharide complexes to address the “shelf-life vs. bioavailability” dilemma of polyphenols in food applications

**DOI:** 10.3389/fnut.2025.1733545

**Published:** 2025-12-17

**Authors:** Shen Qu, Hua Bai, Yan Xu, Borui Li, Xinna Wang

**Affiliations:** 1 Anhui Huatuo Institute of Traditional Chinese Medicine, Bozhou, China; 2 Affiliated Hospital of Changchun University of Chinese Medicine, Changchun, China

**Keywords:** protein-polysaccharide complexes, polyphenols, complex coacervation, layer-by-layer, controlled release, bioavailability, clean label

## Abstract

Polyphenols exhibit significant potential as functional food ingredients owing to their pronounced health-promoting effects. However, their practical application is substantially limited by a central technological dilemma: the trade-off between chemical instability during food processing and storage, and low bioavailability within the human gastrointestinal tract. This review focuses on a nature-derived, biocompatible, and sustainable strategy to address this challenge, which involves the use of molecular self-assembly between proteins and polysaccharides to construct nano- or micro-scale delivery systems. We systematically elucidate how these natural biopolymer complexes, through their sophisticated structural designs, offer a promising solution to mitigate the aforementioned dilemma. The review provides an in-depth analysis of two core fabrication mechanisms—complex coacervation and layer-by-layer (LbL) electrostatic deposition. It highlights how these structures, via functional stratification, not only provide a physical barrier to protect polyphenols from environmental stressors in complex food matrices but also confer the ability for smart release in response to specific physiological signals in the digestive tract, such as pH shifts and enzymatic digestion. Finally, we assess the feasibility and challenges of translating this technology from the laboratory to industrial-scale production, emphasizing its unique advantages in developing “clean label” functional foods. Future perspectives on precise structural control, multifunctional co-delivery, and interactions with gut microbiota are also discussed.

## 1 Introduction

Although polyphenol-rich “superfoods” such as blueberries and green tea are highly acclaimed, their purported health benefits, including potent antioxidant and anti-inflammatory effects (1), are often substantially diminished during industrial food production, extended shelf-life, and complex human digestion. This prevalent “value loss” represents a critical challenge in the functional food sector. On one hand, the phenolic hydroxyl groups in the molecular structure of polyphenols render them extremely sensitive to environmental factors like oxygen, light, metal ions, and pH changes, leading to rapid degradation and loss of biological function during processing and storage (2, 3). On the other hand, even if polyphenols are consumed intact, their low water solubility, limited absorption by the gastrointestinal barrier, and rapid metabolism by the gut microbiota collectively result in generally low bioavailability in the human body (4).

To address this challenge, researchers have developed various encapsulation technologies, such as liposomes, nanoemulsions, and cyclodextrins. However, while seeking a balance between extending shelf-life and enhancing bioavailability, these technologies also face significant considerations related to cost, stability, regulation, or consumer acceptance, which will be critically evaluated. This review, therefore, aims to first provide a deep analysis of the core polyphenol dilemma. We will then critically assess the specific limitations of mainstream conventional technologies. Finally, we will systematically expound the fabrication philosophy and functional mechanisms of an alternative, nature-inspired strategy—protein-polysaccharide complexes—as a promising “clean label” solution to these established challenges.

## 2 The origin of the dilemma: a microscopic perspective on molecular interactions

A profound understanding of the “shelf-life vs. bioavailability” dilemma requires a molecular-level analysis of the complex challenges polyphenols face in two key scenarios: the food matrix and the human gastrointestinal tract.

### 2.1 Chemical degradation and physical instability in the food matrix

The food matrix is a complex chemical reaction system. Once introduced, the phenolic hydroxyl groups in polyphenol structures become active sites for various degradation reactions. First, the presence of dissolved oxygen is a primary cause of oxidation, a process significantly accelerated by the catalysis of metal ions (e.g., Fe^3+^, Cu^2+^). This leads to the conversion of polyphenols into quinones, which subsequently undergo polymerization or cleavage, ultimately resulting in the loss of antioxidant activity and undesirable color changes (e.g., browning) (5). Second, light, particularly ultraviolet radiation, can provide the energy to initiate photodegradation reactions, destroying the fundamental skeleton of polyphenols. Furthermore, pH fluctuations in the food system are a critical influencing factor. Changes in pH not only affect the ionization state and solubility of polyphenol molecules but may also directly catalyze certain hydrolysis or isomerization reactions, leading to physical instability, such as precipitation from the solution or non-specific binding and aggregation with other macromolecules, thereby affecting product homogeneity and texture (6).

### 2.2 Biological barriers and microbiota metabolism in the gastrointestinal tract

**Figure 1:**
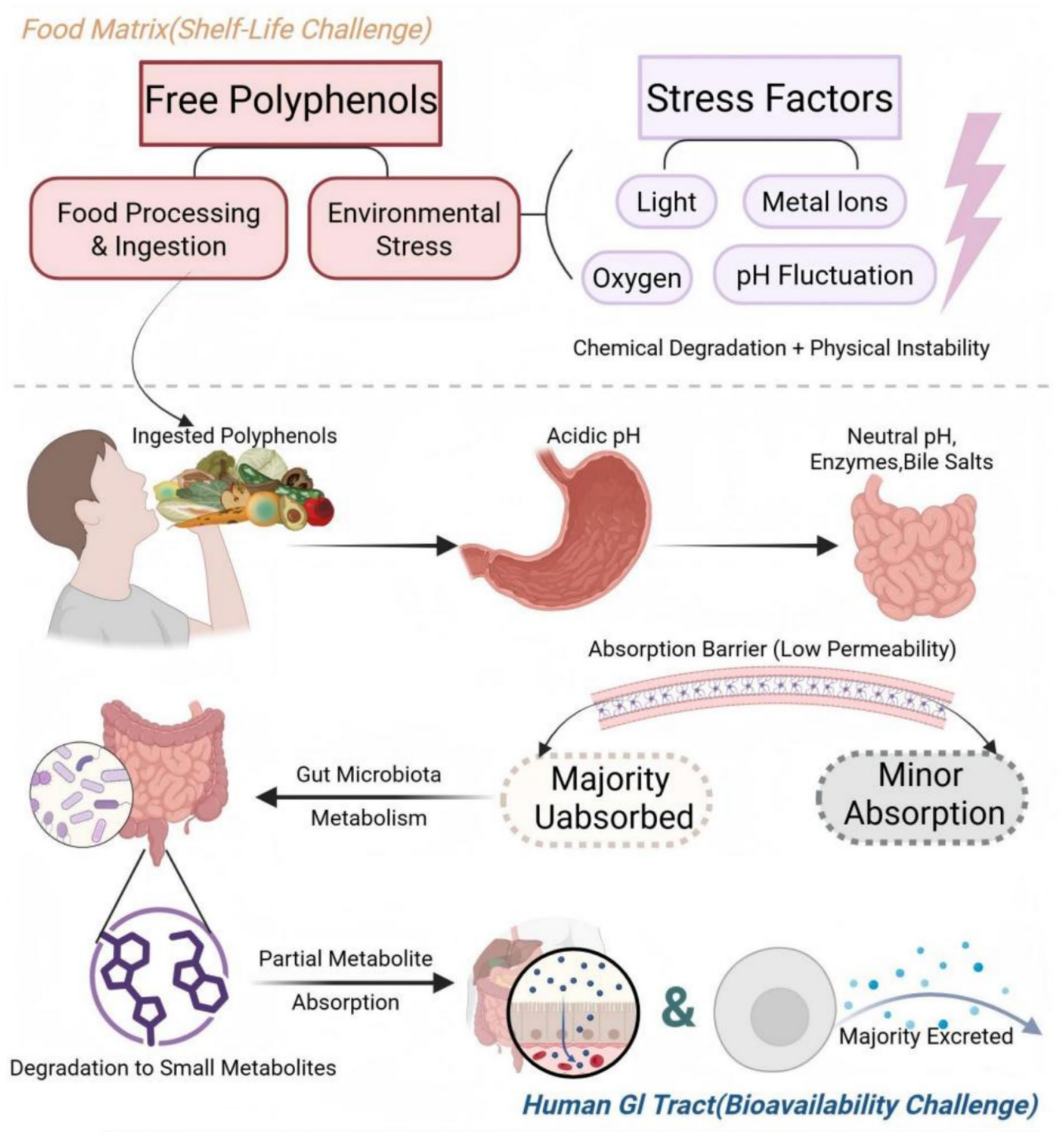
This diagram illustrates the challenges faced by polyphenols in two core scenarios.

When polyphenols enter the human gastrointestinal tract, the challenge shifts from a chemical to a more complex biological environment. In the oral cavity, polyphenols may interact with macromolecules such as proline-rich proteins in saliva, forming complexes that can affect their subsequent dissolution and absorption. Upon entering the stomach, the highly acidic environment (pH 1–3) tests the stability of certain polyphenols (e.g., anthocyanins), but the main absorption barrier occurs in the small intestine. The neutral environment of the small intestine, along with the presence of bile salts and digestive enzymes, alters the dissolution state and chemical form of polyphenols. More importantly, the lipid bilayer structure of the small intestinal epithelial cell membrane presents a significant permeability barrier to the more polar polyphenol molecules. Only a few small, relatively lipophilic polyphenols can be effectively absorbed via passive diffusion or active transport (3). The majority of unabsorbed polyphenols proceed to the colon, where they encounter the body’s largest and most complex microbial community—the gut microbiota. Trillions of bacteria in the colon produce a wide variety of enzymes that can degrade complex polyphenols into various small-molecule phenolic acids and metabolites. While this process is a major pathway for polyphenol inactivation, these metabolites may also possess unique biological activities and have profound effects on host health (7), as shown in Figure 1.

## 3 Conventional encapsulation technologies and their core limitations

To objectively assess the practical value of protein-polysaccharide complexes, they must be placed in a broader technological context and critically compared with other mainstream polyphenol delivery systems, such as liposomes, nanoemulsions, and cyclodextrins. This comparison should not be limited to the single dimension of “clean label” but should encompass multiple aspects, including cost-effectiveness, industrial maturity, technological universality, and core performance.

### 3.1 Liposomes: mature carriers originating from pharmaceutics

Liposomes are spherical vesicles composed of a phospholipid bilayer and have a decades-long history as drug delivery systems, making the technology relatively mature (8, 9). Their core advantage is the ability to encapsulate both hydrophilic (in the aqueous core) and hydrophobic (in the lipid bilayer) polyphenols, with good biocompatibility. However, their application in the food industry faces significant challenges. (1) Cost and Stability Issues: High-quality phospholipids (e.g., soy lecithin, egg yolk lecithin) are expensive, and the liposomal structure itself is sensitive to pH, ionic strength, and shear forces. They are prone to leakage, aggregation, or oxidation in complex food processing environments and during long-term storage, which limits their widespread use. (2) Complexity of Scale-up Production: The preparation of uniform and stable liposomes often requires techniques such as high-pressure homogenization, sonication, or thin-film evaporation. Scaling up these processes is difficult to control and energy-intensive.

### 3.2 Nanoemulsions: high-potential candidates for high loading and bioavailability

Nanoemulsions are thermodynamically unstable systems with droplet sizes below 100 nm, formed by dispersing an oil phase into an aqueous phase using high-energy methods (e.g., high-pressure homogenization, sonication) (10–12). They offer extremely high encapsulation efficiency and loading capacity for hydrophobic polyphenols. Their small particle size provides high optical transparency and potentially high bioavailability (by increasing the specific surface area and promoting lymphatic absorption). However, their disadvantages are equally prominent. (1) High Energy Consumption and Formulation Dependence: Their preparation is a typically high-energy process. Furthermore, the long-term stability of the system is highly dependent on the choice and amount of emulsifier, and many efficient synthetic emulsifiers do not meet “clean label” requirements. (2) Ostwald Ripening: As thermodynamically unstable systems, nanoemulsions undergo Ostwald ripening during storage, where droplet size gradually increases, potentially leading to phase separation. This is a core challenge to their shelf-life stability.

### 3.3 Cyclodextrins: the limitations of a molecular “capsule”

Cyclodextrins are cyclic oligosaccharides composed of glucose units. Their unique “hydrophilic exterior, hydrophobic interior” cavity structure allows them to encapsulate appropriately sized hydrophobic polyphenol molecules, much like a molecular capsule (13, 14). This inclusion significantly improves the water solubility and stability of polyphenols. Their advantages lie in their natural origin, relatively low cost, and ease of use. However, their limitations are also very clear. (1) Selectivity and Loading Limitations: The inclusion action of cyclodextrins has strict requirements for the size and shape of the guest molecule, making it ineffective for all polyphenols. Additionally, one cyclodextrin molecule can typically only include one or a few polyphenol molecules, resulting in a generally low overall loading capacity (w/w). (2) Competitive Displacement: In complex food systems, other small molecules (e.g., flavor compounds, sugars, salts) may compete with polyphenols for the cyclodextrin cavity, leading to premature release of the active substance and loss of protection.

In summary, as a delivery system, the main advantages of protein-polysaccharide complexes lie in the natural origin and low cost of their raw materials, as well as the relatively mild preparation process (self-assembly driven). This makes them highly attractive for “clean label” and sustainable production. However, compared to the aforementioned technologies, they may not have an absolute advantage in terms of loading capacity, precision of particle size control, or stability in certain extreme environments. This limitation in loading capacity, however, is not insurmountable and is an active area of research. Improvements can be achieved through both structural design and process optimization, fully aligning with the “clean label” concept. For instance, in LbL systems designed for hydrophobic polyphenols, the loading capacity is primarily dictated by the primary core; process improvements therefore focus on creating high-payload primary emulsion cores before stabilization by the LbL shell. For complex coacervation, loading can be enhanced by optimizing process parameters (e.g., pH, polymer ratio) to maximize the polyphenol’s affinity for, and partitioning into, the coacervate phase. Furthermore, advanced structural designs, such as forming porous hydrogel particles or nanogels, allow for subsequent loading via diffusion, potentially increasing the payload. Even modifying the biopolymers themselves, for example through controlled thermal treatment to expose more hydrophobic binding sites on proteins, represents a promising strategy to enhance binding and loading of specific polyphenols. Therefore, the choice of technology should be based on a comprehensive assessment of the specific application scenario, cost budget, and regulatory requirements. Collectively, these limitations highlight a clear technological gap: the need for a delivery system that is simultaneously cost-effective, derived from “clean label” sources, scalable, and capable of smart, responsive release.

## 4 A nature-inspired solution: fabrication and function of protein-polysaccharide complexes

To address the specific technological gaps—such as high cost, synthetic components, and stability trade-offs—identified in Section 3, a strategy utilizing natural biopolymers offers a compelling, “clean label” alternative. The construction of protein-polysaccharide complexes is essentially an art of harnessing and orchestrating intermolecular non-covalent interactions. By precisely controlling the external environment, these biological macromolecules can be guided to self-assemble into supramolecular systems with specific structures and functions. Among them, two core technologies based on electrostatic attraction—complex coacervation and LbL electrostatic deposition—are the most extensively studied and mature strategies.

### 4.1 Core fabrication mechanisms: harnessing intermolecular forces

Complex coacervation is a typical liquid–liquid phase separation phenomenon. Bioactive substances like polyphenols can be effectively encapsulated and enriched within the coacervate phase before or during its formation, which is subsequently solidified into microcapsules by methods such as cross-linking or drying (15, 16). For instance, under acidic conditions (e.g., pH 4.6), whey protein isolate (WPI), which is positively charged below its pI (≈ 5.1), interacts with peach gum (PG), which is negatively charged above its pKa, to form dense coacervate microcapsules (17). These have been used to encapsulate sweet orange essential oil and successfully applied to preserve fresh-cut pears (15). The efficiency of this process and the properties of the final product are extremely sensitive to parameters such as pH, ionic strength, polymer type, molecular weight, charge density, and the mass ratio of the two polymers. Precise control of these variables is key to achieving high encapsulation efficiency and optimizing product performance.

LbL electrostatic deposition offers a more refined and controllable fabrication method. This technique uses a pre-fabricated nano- or micro-sized particle with a surface charge as a template core. By alternately depositing oppositely charged protein and polysaccharide solutions onto the core’s surface, an “onion-like” multilayer shell structure is built up layer by layer (6). This core template can be an emulsion oil droplet encapsulating a lipophilic polyphenol or a polyphenol-loaded nanoparticle made of protein itself. With each layer deposition, the particle’s surface charge is reversed, providing the driving force for the adsorption of the next oppositely charged polymer. For example, treating a negatively charged oil droplet with a positively charged chitosan solution forms the first protective layer; after washing away excess chitosan, a negatively charged sodium alginate solution is used to form the second layer, and so on (18). The unique advantage of the LbL technique lies in its high designability. Researchers can precisely control the number, thickness, sequence, and composition of the shell layers, thereby enabling fine-tuning and programming of the delivery system’s stability, permeability, and release behavior (19).

Indeed, the selection between complex coacervation and LbL electrostatic deposition is significantly guided by the physicochemical properties of the target polyphenol, most notably its polarity and solubility. Complex coacervation is generally more suitable for hydrophilic or water-soluble polyphenols. As these molecules are already dissolved in the aqueous phase, they can be directly captured and enriched into the dense, polymer-rich coacervate phase as it forms, resulting in a “matrix-type” encapsulation. The encapsulation efficiency and stability in this system depend not only on the density of the coacervate but also on the non-covalent affinities (e.g., hydrogen bonding, hydrophobic interactions) between the polyphenol and the polymer matrix. In contrast, the LbL technique is the ideal choice for encapsulating hydrophobic or lipophilic polyphenols (such as curcumin or essential oils). Given their insolubility in water, these compounds must first be dissolved within a “core,” typically an oil droplet in a primary emulsion. The LbL technique is then employed to build a structurally precise, multilayered shell around this pre-formed core by alternately depositing oppositely charged proteins and polysaccharides. Consequently, its encapsulation efficiency is primarily determined by the initial payload of the core, while its structural stability relies on the number, integrity, and thickness of the shell layers.

### 4.2 Functional realization I: a “robust shield”—multidimensional resistance to environmental stress

The primary function of protein-polysaccharide complexes, constructed via complex coacervation or LbL assembly, is to provide a robust physical barrier for the encapsulated polyphenols. This barrier effectively isolates them from various external stressors, thereby maintaining their chemical stability and biological activity during the product’s shelf-life (20). For example, numerous *in vitro* studies have confirmed that the encapsulation of highly sensitive polyphenols, such as curcumin or EGCG, within these complexes significantly slows their degradation under harsh conditions like UV light or oxidative stress, often retaining >90% of the polyphenol compared to <50% for the free counterparts (21).

First, in terms of antioxidation, the dense biopolymer network structure plays a crucial role in isolation. It can significantly reduce the permeation rate of oxygen, free radicals, and catalytically active metal ions into the core region, thus effectively inhibiting oxidation reactions (22). Some studies have further enhanced this protective effect by introducing polyphenols that themselves possess strong metal ion-chelating and free-radical-scavenging abilities, such as tannic acid, as cross-linkers or structural units. For example, using metal-phenolic networks (MPNs) based on tannic acid to harden the surface of coacervate microcapsules not only enhances the physical strength of the structure but also endows the wall material with intrinsic antioxidant capacity, creating a dual-protection mechanism of “passive isolation + active scavenging” (5, 23).

Second, regarding resistance to photodegradation, the protein and polysaccharide molecules themselves contribute significantly. The chemical structures of some polysaccharides and polyphenols (e.g., ferulic acid, gallic acid) also enable them to absorb or scatter UV light. When these molecules are densely packed in the shell of the complex, they collectively form a natural “sunscreen layer” that can effectively reduce the probability of high-energy photons penetrating and damaging the core polyphenol molecules (24).

Finally, in maintaining pH stability, the dense multilayer structure or coacervate phase provides a relatively independent microenvironment for the encapsulated polyphenols, buffering them from the impact of extreme pH values in the external food matrix. Whether in acidic beverages (pH 2.5–4.0) or in some neutral or even slightly alkaline food systems, this physical isolation can effectively prevent the core substance from undergoing hydrolysis, changes in ionization state, or precipitation due to drastic pH shifts. This greatly broadens the range of product categories and application scenarios for polyphenol-containing functional foods (25).

### 4.3 Functional realization II: a “smart key”—sequential release in response to digestive signals

If a robust physical barrier solves the shelf-life problem, then the delivery system’s ability to intelligently respond to environmental signals within the gastrointestinal tract is the key to unlocking the bioavailability challenge. The ingenuity of protein-polysaccharide complexes lies in their dynamically tunable structural stability, allowing them to function like a “smart key” that is unlocked sequentially at different stages of the digestive journey.

In the stomach environment (pH 1–3), most proteins carry a substantial positive charge, while many acidic polysaccharides (e.g., pectin, sodium alginate) have a high degree of carboxyl group protonation and thus less negative charge. However, the electrostatic attraction between them is usually sufficient to maintain a relatively compact and stable structure. This tight structure effectively protects the internal polyphenols from degradation by gastric acid and prevents their premature release and inactivation. This acid-stability has been demonstrated in systems protecting anthocyanins, which would otherwise rapidly degrade and lose color in low-pH gastric environments, showing minimal release (<10%) in simulated gastric fluid (26). The chemical basis of this protective mechanism shares commonalities with the strategy used by mussel adhesive proteins, where catechol structures maintain redox homeostasis in a variable marine environment; both rely on physical isolation or chemical microenvironment modulation to protect highly reactive phenolic hydroxyl structures (27). Ultimately, this ensures that the majority of the active substance arrives safely at its primary absorption site—the small intestine.

When the chyme enters the small intestine (pH 6.5–7.5), the environment changes significantly, triggering the “unlocking” mechanism of the complex. The first trigger is pH response: as the pH increases, the carboxyl groups on the polysaccharide molecules become extensively deprotonated, leading to a sharp increase in negative charge density. Concurrently, the positive charge on the protein decreases. This results in a substantial weakening of the electrostatic attraction between them, which may even turn into electrostatic repulsion, causing the complex network structure to swell, loosen, or even disintegrate (3). The second trigger is enzymatic response: the small intestine is rich in proteolytic enzymes such as trypsin and chymotrypsin, which specifically attack and degrade the protein backbone of the complex, further compromising its structural integrity. The synergistic action of these two mechanisms allows the encapsulated polyphenols to be released in large quantities, creating a high local concentration that facilitates subsequent transmembrane absorption.

For delivery systems constructed with a backbone of digestion-resistant polysaccharides, the journey is not over. These polysaccharide backbones can resist enzymatic hydrolysis in the upper gastrointestinal tract, escorting a portion of unabsorbed polyphenols or specifically designed colon-targeted active substances to the colon. To achieve this, the primary challenge is to bypass the release triggers of the small intestine. This requires careful material selection and structural design. The polysaccharide backbone (e.g., pectin, inulin, or guar gum) must be specifically chosen for its known resistance to host enzymes (like pepsin and trypsin) while being a substrate for microbial enzymes (glycosidases) in the colon. Furthermore, to prevent the structure from swelling and disintegrating at the neutral pH of the small intestine, its integrity must be enhanced. This is often achieved through cross-linking (e.g., using “clean label” cross-linkers like tannic acid) to “lock” the polymers together, or in an LbL design, by using a robust, digestion-resistant polysaccharide as the outermost protective layer to shield inner protein components from proteolytic attack. In the colon, the vast gut microbiota secretes a variety of glycosidases, which the host lacks, to ferment and degrade these polysaccharide backbones. This process not only achieves the final, targeted release of the active substance in the colon—allowing it to directly modulate the composition and metabolism of the gut microbiota or treat local colonic diseases—but the degradation products of the polysaccharides themselves also exert significant prebiotic effects, creating a synergistic enhancement of bioactivity with the polyphenols (28). This synergy operates through multiple pathways. For example, the prebiotic polysaccharide (e.g., inulin, pectin) selectively promotes the growth of beneficial bacteria (such as Bifidobacteria and Lactobacilli), which are often the primary microbes responsible for metabolizing the polyphenols into their more bioactive small-molecule metabolites. Furthermore, the fermentation of these polysaccharides produces short-chain fatty acids (SCFAs) like butyrate, which possess potent anti-inflammatory properties that can act in concert with the anti-inflammatory effects of the polyphenol metabolites, enhancing overall gut health and barrier function.

**Figure 2:**
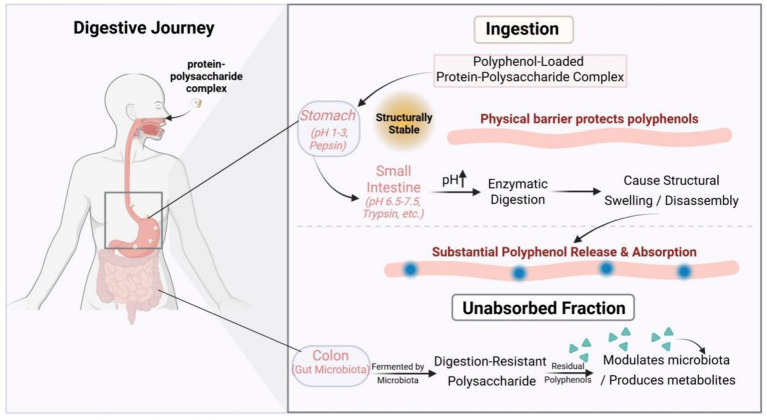
This diagram depicts the “digestive journey” of a protein-polysaccharide complex as a smart delivery system.

However, it is noteworthy that current assessments of this “smart release” behavior largely rely on simplified, static *in vitro* digestion models. Although these models provide convenience for preliminary screening and mechanistic exploration, their simulated environment differs significantly from the complex dynamic processes within the actual gastrointestinal tract (e.g., peristalsis, real-time interaction between enzyme concentrations and substrates). Therefore, caution must be exercised when extrapolating from the release profiles observed in these *in vitro* experiments to predict actual bioavailability enhancement *in vivo*. The definitive efficacy awaits further validation by more advanced dynamic digestion systems or in vivo studies, as shown in Figure 2.

## 5 From “ideal design” to “industrial reality”: unique advantages and challenges of the natural approach

Although protein-polysaccharide complexes show great potential in theoretical design, translating them from ideal laboratory models to scalable industrial reality requires an objective assessment of their inherent advantages and the challenges they face.

### 5.1 Advantages: a clear path to “clean label”

The greatest appeal of this technology is its high compatibility with the current food industry trend toward “clean label.”

First, from a regulatory and ingredient perception standpoint, most of the proteins (e.g., whey protein, casein, gelatin) and polysaccharides that constitute the carriers are GRAS food ingredients. They do not require the lengthy and stringent toxicological approvals needed for synthetic polymers, which significantly reduces the regulatory barriers and time-to-market for new products (1). In terms of marketing and consumer acceptance, terms like “whey protein” and “pectin” are familiar, natural, and perceived as harmless by consumers, making them easy to communicate and accept, thus avoiding consumer skepticism that can arise from complex chemical names.

However, it is worth noting that the concept of “clean label” is multidimensional, concerning not only the natural origin of ingredients but also the “simplicity” of the processing. Although complex coacervation and LbL assembly are based on spontaneous molecular behavior, their industrial implementation may involve a series of complex physical and chemical processing steps, such as drastic pH adjustments, high-speed shearing, centrifugation, and spray drying. Whether this “highly engineered” processing still fully aligns with some consumers’ intuitive perception of “minimally processed” and “all-natural” is a market issue worth exploring. Therefore, product communication needs to skillfully convey the “nature-derived” attribute of the ingredients while providing a reasonable explanation for the necessity of the processing.

Finally, the technology also has potential multifunctionality. Many polysaccharides used as wall materials are themselves functional ingredients, such as dietary fibers or prebiotics, which can promote gut health. This means the carrier not only protects and delivers polyphenols but can also create a synergistic health benefit with the core active ingredient, providing additional functional claims for the product.

### 5.2 Challenges: process control from “beaker” to “reactor”

However, scaling up this sophisticated molecular self-assembly process from the laboratory “beaker” to the industrial “reactor” brings a series of process control challenges to the forefront.

First, process sensitivity and uniformity control. Both complex coacervation and LbL assembly are highly sensitive to numerous parameters, including pH, temperature, ionic strength, polymer concentration, and mixing rate and order. Conditions that can be precisely controlled on a small scale become exceptionally difficult to maintain uniformly and consistently between batches in large-scale production. For example, non-uniform mixing rates in a large reactor can lead to local deviations from optimal polymer concentrations or pH, resulting in the formation of large, irregular aggregates instead of ideal microcapsules. This can lead to a wide particle size distribution and significant batch-to-batch variation in encapsulation efficiency (15). To mitigate this, introducing online process analytical technology (PAT) is a critical future direction. Real-time monitoring of key parameters, such as in-situ particle size analysis (e.g., via DLS probes) or Zeta potential, could provide immediate feedback for adaptive control of mixing speeds, pH titration rates, or polymer dosing, thereby ensuring greater uniformity.

Second, the challenge of raw material standardization. Unlike chemically synthesized polymers, the physicochemical properties of natural biopolymers are subject to inherent variability. For example, gum arabic from different harvest seasons may have significant differences in protein content and polysaccharide chain branching, which directly affects its performance as an emulsifier and wall material. Similarly, pectin obtained through different extraction processes will have varying degrees of methylesterification and molecular weight distributions, which directly determines the optimal conditions for its coacervation with proteins at a specific pH. This raw material uncertainty requires manufacturers to establish strict quality control standards for raw materials and corresponding process adjustment protocols, which undoubtedly increases production costs and complexity.

Third, the impact on the final product’s sensory properties. As macromolecules, the addition of proteins and polysaccharides to food inevitably affects its sensory attributes. A typical example is in clear, acidic beverages. The addition of protein-polysaccharide complex particles, even at the nanoscale, often causes undesirable turbidity or opalescence due to light scattering. Furthermore, these macromolecules can significantly increase the viscosity of the liquid, altering the product’s “mouthfeel.” A more challenging issue is that certain proteins may introduce beany off-flavors, while some polysaccharides can impart an astringent sensation. To address this, formula adjustments can be made, such as selecting raw materials with a more neutral sensory profile (e.g., substituting beany-tasting plant proteins with blander whey or pea isolates) or utilizing lower molecular weight polymers to reduce the impact on viscosity. Process optimizations offer further solutions; for instance, reducing the particle size of the complexes (e.g., via high-pressure homogenization) to well below the wavelength of visible light can maintain optical clarity in beverages. Additionally, processing steps such as de-flavoring the protein or polysaccharide raw materials before forming the complex can be employed. How to achieve effective encapsulation while minimizing negative impacts on the original flavor and texture of the product through careful formulation design (e.g., adding flavor maskers) or process optimization is a critical trade-off that formulators must carefully consider and resolve.

Fourth, commercialization barriers beyond the technology itself. From an economic perspective, despite the “clean label” advantage, the overall cost-effectiveness of this technology must be compared with existing mature solutions. For instance, compared to the direct addition of inexpensive synthetic antioxidants or the use of lower-cost cyclodextrin inclusion technology, the production of protein-polysaccharide complexes involves more complex process control and higher raw material quality control costs. Its commercial success depends on whether the added value of the final product is sufficient to cover this premium and be accepted by consumers. For small and medium-sized enterprises with limited capital and R&D capabilities, establishing such a complex production and quality control system may constitute a significant barrier to entry. From a supply chain perspective, the “variability” of natural raw materials is not just a technical challenge but also a supply chain management problem. This requires downstream food companies to establish deeper strategic partnerships with upstream raw material suppliers to jointly develop and implement strict raw material acceptance standards, and even extend upstream to participate in the cultivation or primary processing of raw materials to ensure supply chain stability and product quality consistency.

### 5.3 Future product concepts for functional foods

While acknowledging these industrial challenges, the technology’s potential, as demonstrated by current laboratory-scale successes, points toward a new generation of more advanced functional food products. These future concepts are not mere speculation but are logical extensions of the structure–function relationships already discussed.

First, the technology holds the promise of creating the first truly “high-concentration, high-stability, all-natural” clear polyphenol functional beverage. By encapsulating polyphenols in carefully designed nanocomplexes with particle sizes far smaller than the wavelength of visible light, the problems of turbidity and precipitation caused by polyphenol addition can be effectively solved without the use of any synthetic emulsifiers or stabilizers. This would enable the development of “super waters” or functional sparkling waters that are as clear as water, have a refreshing taste, and are rich in high doses of stable polyphenols.

Second, the advent of “functional popping pearls” can be envisioned. Micron-sized capsules prepared by complex coacervation can be designed to be stable during shelf-life but rupture instantaneously in the mouth in response to specific temperatures, pH, or enzymes. These “popping pearls” could be added to yogurt, ice cream, or breakfast cereals, not only bringing a novel texture and eating experience but also releasing fresh, highly active polyphenols or flavor substances at the moment of consumption, perfectly combining sensory stimulation with health benefits.

Finally, and most disruptively, is the construction of a “synbiotic enhancer” that can simultaneously carry probiotics and targeted polyphenols. This sophisticated multilayer structure could protect probiotics in its core, with an outer layer encapsulating prebiotics “customized” for them and polyphenols for modulating the gut environment. This system would act like a “landing craft,” protecting the probiotics from gastric acid and bile salts to safely reach the colon. Upon arrival, the outer structure would degrade in response to the colonic environment, simultaneously releasing prebiotics and polyphenols to create an optimal microenvironment for the colonization and proliferation of the probiotics. This would achieve a “precision fertilization” type of targeted intervention in the gut microbiome, maximizing health benefits.

## 6 Conclusion and future perspectives

### 6.1 Conclusion

In addressing the core challenge of chemical instability and low bioavailability of polyphenols in functional food development, returning to nature and utilizing endogenous food components to construct smart delivery systems has emerged as a highly promising approach. This review has systematically elucidated how nano/micro-scale complexes, fabricated through the self-assembly of proteins and polysaccharides (particularly via complex coacervation and LbL electrostatic deposition), are designed to synergistically achieve the dual functions of “physical barrier protection” and “physiological signal-responsive release.” This strategy offers a promising solution to the “shelf-life vs. bioavailability” dilemma. More importantly, its complete reliance on natural, safe, food-grade raw materials aligns perfectly with the current market demand for “clean label” products, giving it unique application potential among various delivery technologies. From a broader perspective, the technological path discussed in this review is not just a specific solution within food science but also an excellent embodiment of the grander scientific vision of “Nature-Inspired Smart Material Design.” Its core design philosophy—utilizing the intrinsic interactions between biological macromolecules for bottom-up, ordered construction—is conceptually analogous to cutting-edge fields such as controlled drug release systems in biomedicine and self-healing hydrogels in materials science. This strategy of translating the elegant construction principles evolved by nature over billions of years into solutions for real-world engineering problems demonstrates the immense potential of interdisciplinary research and provides valuable inspiration for the future development of more intelligent and sustainable materials that are derived from, and may even surpass, nature. It should be noted that this review primarily focuses on protein-polysaccharide complexes based on electrostatic interactions, with less emphasis on delivery systems constructed through other forces or other strategies. Future review work could complement this field from a broader perspective.

### 6.2 Future research directions

Although significant progress has been made, this field still has vast room for exploration. Future research should focus on the following directions to advance the technology to deeper levels and broader applications.

First, a shift from “bulk mixing” to “micro-scale precision fabrication.” Most current research still employs conventional bulk mixing methods to prepare complexes, which has limitations in controlling the uniformity and structural reproducibility of the product. In the future, precision manufacturing technologies such as microfluidics and electrospinning should be increasingly incorporated to achieve precise control over the size, morphology, and shell thickness and number of the complexes at the single-particle level. This will help establish a clearer structure–property relationship between the structural parameters and release kinetics.

Second, an upgrade to “multifunctional co-delivery platforms.” Besides delivering polyphenols, this system can be used to co-encapsulate other functional ingredients to achieve synergistic effects. A highly promising direction is the design of composite delivery systems that can simultaneously encapsulate polyphenols and specific probiotic strains or prebiotics. Such a system would not only protect the probiotics during their transit through the gastrointestinal tract but also enable the synchronous release of polyphenols and prebiotics in the colon. This would achieve “precision fertilization” and “targeted intervention” of the gut microbial environment, maximizing its health benefits for the host. Third, a deeper focus on regulating metabolite production. Future structural design should not only target if a polyphenol is released, but how. By precisely controlling the release kinetics (e.g., fast vs. slow release) and the specific site of release within the colon (proximal vs. distal) through structural modulation (e.g., cross-linking density, LbL layer composition), it may be possible to guide the polyphenol’s metabolism toward specific, highly bioactive pathways. This involves understanding how varying substrate concentrations and co-delivered prebiotics influence the competitive metabolism by different microbial consortia. Fourth, a deepening of research methods toward more realistic *in vivo* simulations. Future research urgently needs to incorporate more advanced dynamic *in vitro* digestion systems (such as TNO’s TIM-1) and even in vivo animal models. These tools will allow for the tracking and study of the complex behaviors, degradation pathways, and metabolite profiles of these complexes under simulated real-world conditions of digestive peristalsis, dynamic enzyme concentrations, and interactions with the food matrix. This will provide more reliable and instructive scientific evidence for the structural optimization and functional validation of these delivery systems.
